# Back to basics: Gaps in baseline data call for revisiting an environmental education program in the SAVA region, Madagascar

**DOI:** 10.1371/journal.pone.0231822

**Published:** 2020-04-21

**Authors:** Marina B. Blanco, Alexie N. Rudman, Lydia K. Greene, Fusiane Razafindrainibe, Lanto Andrianandrasana, Charles Welch

**Affiliations:** 1 Duke Lemur Center, Durham, NC, United States of America; 2 Nicholas School of the Environment, Duke University, Durham, NC, United States of America; 3 University Program in Ecology, and Department of Evolutionary Anthropology, Duke University, Durham, NC, United States of America; 4 Duke Lemur Center-Sava Conservation Initiative, Sambava, Madagascar; University of Waikato, NEW ZEALAND

## Abstract

Environmental education programs are critically important for raising awareness about global and local environmental concerns, by providing the knowledge, tools, and means for young and old generations to cope with current challenges. Of the greatest importance is the implementation of environmental education programs in biodiversity hotspots where environmental crises are high and resources to fund mitigation programs are low. Madagascar is one such priority hotspot, featuring intensive wildlife-human conflicts due to shrinking natural environments. The Duke Lemur Center-SAVA Conservation Initiative has been conducting environmental education activities in the SAVA region, northeastern Madagascar, since 2011. These activities have been aimed at increasing awareness of local and global environmental issues and at stimulating the involvement of local school students. Our initiatives have predominantly supported teacher trainings to provide teachers with environmental education content, examples, and recommendations on how to integrate activities into their regular curricula, commonly referred to as a “cascade approach”. Due to logistical hurdles, including high teacher turnover rates, however, these interventions have not been monitored to assess their efficacy. Thus, to better inform current and future interventions, we designed and implemented classroom-based surveys to gather baseline data on the basic knowledge of SAVA students and their perceptions of the environment. We identify informational gaps in surveyed schools, including those located in large towns vs. villages, and those with trained vs. untrained teachers. Based on these results, we make recommendations for future environmental education efforts in the region, including activities that could address real-life environmental problems and decision-making solutions.

## Introduction

The development and implementation of environmental education (EE) programs is more imperative than ever. We live in a world that is changing at a rapid pace, in which the “rules of nature” are bent by human recklessness across global ecosystems [1]. EE programs play a crucial role in raising awareness about environmental concerns and incentivizing behavioral changes to address these issues. These programs are particularly relevant in biodiversity hotspots, where environmental crises are rampant, and where population growth rates are higher than the world’s average, increasing the prevalence of wildlife-human conflicts [2]. Different approaches coexist under the umbrella of Environment and Education programs, some attempting to address the integration of human needs with sustainability, others targeting environmental problems by providing knowledge, skills, and tools for active participation [3].

A “cascade approach” has been commonly used in EE programs around the world, because it can be cost-effective and successful, if certain criteria are respected [4]. In cascade-based EE programs, school teachers are usually in charge of conducting EE activities, because of their standing in the community as authority figures, and their roles as communicators and mentors. Each teacher has the potential to reach hundreds of students at once and spread information at an exponential pace. Despite these clear advantages, however, shortcomings to this approach are common and pose real threats to the success of these programs [5,6].

In Madagascar, the contrast between complex biodiversity-rich environments, on the one hand, and human populations relying on natural resources for survival, on the other, could not be any sharper. Poverty and low levels of educational attainment challenge the ability of teachers to incorporate environmental education topics in school. Although the cascade approach has been frequently implemented in Madagascar, teachers, especially in rural areas, often struggle with a lack of basic resources (blackboards, chalk, and other class materials), as well as with the knowledge and/or confidence to incorporate EE activities into their curricula [7–9]. This is partly the result of traditional training in a culture reliant on fact citation, without providing critical thinking skills to students [3].

Despite being immersed in a traditionally-framed education system, a new generation of Malagasy teachers has been encouraging more active student participation. Examples of more dynamic and engaging activities include multi-media approaches, problem-solving activities, and other active learning techniques, known to bring positive impact [10–12]. Many of these renewed strategies have been designed, financed, and implemented with significant support from international programs, acting alone or in concert with national organizations [9]. Foreign intervention in developing countries has been criticized on cultural levels, e.g., for a lack of sensitivity or understanding of local people’s cosmology and beliefs, and their complex relationship with the land. In particular, this can happen when foreigners impose “western” views on traditional practices [13]. However, international programs can be greatly beneficial in providing resources, such as funding opportunities, otherwise unavailable to the target country. Foreign views may also provide different perspectives about sustainability, conservation, and development, thereby broadening the horizon of local participants who get the chance to appreciate their country’s riches from a different perspective [8].

Although the last several decades have been witness to an increasing number of EE programs, they are often implemented without first gauging the students’ baseline understanding of environmental issues. Without such data, coupled with quantitative or qualitative evaluations of these programs (which are generally lacking), researchers are prevented from testing whether their goals have been partially or fully achieved. A lack of accountability has also prevented data collection that is needed to legitimize the program to the responsible agencies, including potential donors [14,15,9]. Monitoring EE programs is challenging for a variety of reasons, chief among them including a lack of funding (i.e., it is more exciting to implement a program and count participants than to invest in follow-up impact reports, which may be challenging to execute in many programs), a lack of trained staff to conduct the sampling, and importantly, a lack of simple strategies to assess success and link responses/actions to specific activities. There are also temporal effects that are hardly discussed: Immediately after the implementation of a program, there is a spike of interest and motivation as a result of novelty and promise. Whether behavioral changes and long-term impact are sustained over prolong periods of time is more difficult to prove and, consequently, less reported overall.

The Duke Lemur Center-SAVA Conservation Initiative (DLC-SAVA), based in the coastal town of Sambava, northeastern Madagascar, has been conducting community-based conservation activities since 2011. As part of its EE program, the DLC-SAVA has been supporting teacher trainings, a program originally developed by the Madagascar Flora and Fauna Group in the 1990’s at Ivoloina, a conservation and nature center in eastern Madagascar [16]. For the DLC-SAVA-supported teacher trainings, local teachers are recruited by the Regional School Office authorities (Circonscription Scolaire) at a centralized office location to undergo a 5-day intensive workshop. During the training, participating teachers are presented with a “Manual” (Guide Pratique du Maitre) containing EE content (e.g., lesson plans, practical exercises) and tools to integrate environmental topics in pre-existing school subjects, such as geography, science or history. The current manual, written in French, was developed in the 1990’s by local educators and Madagascar Fauna Group staff and had the approval of the Ministry of Education to be used in Malagasy schools [16]. Through the Manual, teachers can read about a variety of concepts and themes: e.g., ecosystems, climate, air and water cycles, etc., as well as obtain information regarding environmental problems such as soil degradation and water contamination, among others (for more information visit https://www.madagascarfaunaflora.org/environmental-education.html).

Teacher trainings have been a successful component of the DLC-SAVA program, if success is measured by the number of teachers trained in the SAVA region. Over the last four years, the program has supported the training of more than 1,750 teachers belonging to the Sambava and Andapa Districts in the SAVA region. The potential impact of the program at the student level is more difficult to measure. For instance, teachers are encouraged, but not obligated, to incorporate EE content in the curricula; teachers may be inclined to tackle only a subset of issues from the general recommendations; teachers may feel inhibited from using materials referring to general (rather than local) examples such as those cited in the Manual. Finally, we did not gather environmental baseline data across schools prior to implementing teacher trainings, which precludes us from any evaluation efforts thus far.

Therefore, our study here is focused on gathering baseline data on the students’ perceptions about the environment, information against which we can evaluate ongoing and future EE activities. We designed and implemented classroom-based surveys with the following objectives: (1) to assess and document baseline quantitative data on the students’ environmental knowledge in the SAVA region; (2) to evaluate if the students’ responses differed by their location (city vs. rural villages) and/or by their teacher’s training background (trained vs. untrained, post hoc assessment); (3) to identify informational gaps across students, and thereby be able to better target those issues in future interventions. In this article, we present the results from these surveys, and discuss recommendations for future implementation and monitoring of EE activities.

## Materials and methods

The survey was approved by Duke University’s Human Subjects Institutional Review Board (IRB Protocol #2018–0347). To conduct this research, DLC-SAVA obtained written approval from the Regional School Office authorities (CISCO, Circonscription Scolaire), School Directors and teachers prior to conducting the surveys. The IRB committee waived the need for parental consent and allowed school Directors, acting as guardians, to provide written permission granting our research team to approach school children about participation in the surveys. Before the commencement of the surveys, the surveyor explained to the students the purpose of the survey and the procedures to follow, assured the students that participation was voluntary, and that no personal information was going to be gathered.

### Study schools and subjects

We designed classroom-based surveys to target students in the last year of primary school. Primary school students were selected for two reasons: (1) Teacher trainings supported by the DLC-SAVA and other organizations target primary school teachers, (2) Only a very small portion of the student population will continue after finishing primary school exams (i.e., less than 3% of students complete secondary level education in Madagascar [9]). Thus, primary school may be the only opportunity for a young student to be exposed to EE activities.

A total of 29 schools (31 classrooms) were surveyed, between March and November 2018 in the SAVA region, northeastern Madagascar. We targeted schools from three Districts: Sambava (13 schools, 11 public, 2 private), Andapa (13 schools, 11 public, 2 private) and Antalaha (3 schools, 1 public, 2 private). The students ranged in age from approximately 12 to 15 years old, and the number of students per classroom varied from 12 to 56, with an average of 38 students. Although it is expected that students in their last year of primary school possess adequate reading and writing skills to fill out a form, during previous activities we have encountered a wide range in writing abilities. To account for possible differences in literacy levels, we designed a survey that relied on photographs and verbally-asked questions and did not require reading or writing.

Surveys were conducted by two Malagasy staff, a “surveyor” (FR) and an “assistant”. Each survey, which took approximately 30 minutes to complete, consisted of three sections (see below; S1 Appendix). Questions were asked verbally in Malagasy and paired with large color photographs (A3 size). The surveyor asked questions while holding up and showing the photographs to all students in the classroom, in a manner that was slow, easy to understand, and unbiased. Every question ended with the sentence “raise your hand if you agree with the statement”, at which point the assistant recorded the affirmative responses in a spreadsheet by tallying raised hands. We should note that although the surveyor requested that students answer the questions *without* looking at any other student, it is possible that some of the students were influenced by the responses of their peers.

### Survey structure

#### Animal section

The purpose of this section was to evaluate whether students could clearly differentiate between domesticated and wild animals. Selected animals were: chicken and dog (domesticated); tenrec, mouse lemur, and brown lemur (wild animals present in the immediate environment); ring-tailed lemur and indri lemur (wild animals absent from the immediate environment). Each survey consisted of three animal choices, randomly selected by student volunteers.

While holding a photograph in front of the classroom the surveyor asked a series of questions: (a) is this animal in the photo a [name with correct answer]? Followed by: is this animal in the photo a [name with wrong answer]? To avoid leading students, the order between correct and wrong answers were randomized among animal choices. This meant that for some animals the first name choice was correct, whereas for others the second name choice was the right one; (b) does [chosen animal] live in a village? Followed by: does [chosen animal] live in a forest? The order of village and forest were randomized among choices; (c) is it acceptable to keep [chosen animal] in a household? (d) is it acceptable to eat [chosen animal]? Finally, while holding a photograph depicting a large lemur, the surveyor asked: (e) does [lemur] need a forest to survive? At no point in the questioning or tallying raised hands were any comments made by the surveyor or the assistant regarding the validity or correctness of the answers.

#### Forest section

The purpose of this section was to assess the students’ general perceptions of the forest, including differentiation of products that can be obtained from the forest, and forest longevity. We were also interested in gathering the students’ responses regarding the value of the forest compared to cultivation land. To begin this section, students were shown a photograph of a “burnt” forest and asked: is this a good forest? Followed by a photo of a primary forest and asked: is this a good forest? There was no affirmation by the surveyor or assistant to whether there was a right or wrong answer. Then, three student volunteers would pick a photograph depicting different articles or materials, such as firewood, a wooden house, a medicinal plant, a traditional lamba (or cloth dress), a cell phone. Once the photograph of a particular item was shown to all students, the surveyor asked: does [chosen article] come from the forest? Finally, the surveyor asked: (a) does a forest take a long time to grow? (b) do trees come from seeds? (c) is a forest as important as a rice paddy?

#### Water section

This section targeted two overlapping issues: the value of water for human consumption and the underlying perception by students about what is considered safe to drink, and whether the students perceived water to be valued for the environment, e.g., for fish and other organisms.

This section begins by showing the students photographs of different sources of water: e.g., a forest stream, a pot of boiled water, a well, water from rice paddies. The surveyor, holding one photograph of a water source at a time asked: is [water source] potable, safe to drink? Once again, there was no statement by the surveyor or assistant to whether some sources of water would be safe for drinking. Next, the surveyor asked: (a) do fish need clean water to survive? (b) do humans need clean water to survive? (c) is it acceptable to throw garbage in the river? (d) is it acceptable to throw poison in the river to catch fish? Finally, the surveyor asked the students what sources of water *they* use at home, by giving them the following examples: water from local river, boiled water, water from a well, purified water. Surveyor asked: do you drink [example] at home? No recommendations were given to students after they answered the questions.

### Statistical analysis

Each classroom was treated as the unit of analysis, with all “yes” responses per question converted to percentages, to account for the variable number of students per classroom. Classrooms were categorized as being led by trained teachers (training) vs. non-trained teachers (no training). It is worth noting that some teachers were exposed to teacher trainings conducted by organizations other than DLC-SAVA. We found no differences if trainings were categorized by institution or combined. Moreover, the average number of years since a training was carried out was 2.75, with some teachers receiving training 7 years ago or a few months before the survey. Thus, “training” here refers to teachers exposed to any EE training program at any point in their past. We further categorized classrooms as belonging to schools in main towns (city) vs. schools near the countryside (village). Median, minimum and maximum values were calculated from classroom percentages. We chose median values, instead of means, because they are less sensitive to extreme outliers in small sample sizes. For statistical analysis, we conducted nonparametric Wilcoxon Mann-Whitney tests in JMP Pro 14.0, to test whether responses across schools differed by training status and/or by school location.

## Results

### Animal section

Overall, the students could accurately identify domesticated animals (chicken and dog) and did fairly well in identifying the wild animals present in their region (tenrec, brown lemur, mouse lemur); however, for the two lemurs classified as wild, but absent from the immediate environment, responses were contrasting: they successfully identified ring-tailed lemurs (which live on the opposite end of the island, in southern Madagascar), but failed to recognize the indri lemur which has an endemic range that overlaps with part of the SAVA region (Table 1). Nevertheless, when students were asked whether the chosen wild animals live in a village or a forest, they almost always raised their hands to answer that they live in a forest, even if they were not able to name the animal.

**Table 1:** List of animals, and responses depicted as medians, minimum and maximum values for animal ID, okay to keep in household, and taboo to eat.

*Category*	*Animal*	*Times selected*	*ID match*		*Household okay*		*Taboo to eat*	
domesticated	dog	15	100	97.7–100	100	80–100	100	76.2–100
domesticated	chicken	10	100	100–100	100	97.7–100	0	0–0
wild present	brown lemur	15	94	0–100	100	0–100	33.9	0–100
wild present	mouse lemur	8	100	86.4–100	88.9	73.7–100	12.1	0–30
wild present	tenrec	15	95.5	66.7–100	2.9	0–94.4	0	0–100
wild absent	indri lemur	17	0	0–86.4	100	88.9–100	90.7	0–100
wild absent	ring-tailed lemur	11	100	66.7–100	77.3	0–100	81.6	0–100

Responses per classroom were converted to percentages.

When the surveyor asked the question of whether it was acceptable to keep the animals in a household, students generally responded “yes” for all animals, domesticated or wild. The tenrec was an exception, scoring low percentages across schools (Table 1). When asked the question of whether it was taboo to eat animals, wild lemurs absent from the immediate area scored higher percentages (i.e., against human consumption) compared to wild animals present in the region. As far as domesticated animals were concerned, dogs showed the greatest taboo against consumption and chickens, unsurprisingly, showed zero (Table 1). A relatively large number of students (median at 32%) agreed with the statement that a lemur can live without a forest. No significant differences were observed among responses (animal ID, acceptable in a household, taboo to eat, lemur without forest) when training status (training vs. no training) or school location (city vs. village) were considered.

### Forest section

In general, the students did not raise their hands when asked if the photo of a burned forest was a “good” forest (0% in 20 out of 29 schools). The students raised their hands when asked if a photo of primary forest was a “good” forest (100% in 24 out of 29 schools). To the questions of whether (a) forests take time to grow, (b) trees come from seeds and (c) forests are as important as rice paddies, students from all schools generally responded affirmatively. There were no significant differences in responses when classrooms were classified by training status. However, when schools were classified as city vs. village, there were significant differences in two instances. Students from village schools scored higher percentages asserting that trees come from seeds (Wilcoxon Mann-Whitney test, *p =* 0.028), and for valuing forests as much as rice paddies (Wilcoxon Mann-Whitney test, *p* = 0.03) (Tables 2 and 3).

**Table 2:** Responses from students when schools are classified by training status.

*Training?*	*#Schools*	*Forest takes time to grow*	*Trees come from seeds*	*Forest as important as rice paddy*
yes	17	100 (81.5–100)	100 (97.1–100)	98 (0–100)
no	12	100 (93–100)	100 (66.7–100)	87.6 (66.7–100)

Median of percentages, minimum and maximum values in parentheses.

**Table 3:** Responses from students when schools are classified by location.

*City school?*	*#Schools*	*Forest takes time to grow*	*Trees come from seeds*	*Forest as important as rice paddy*
yes	12	100 (86.4–100)	98.1 (66.7–100)	83 (66.7–100)
no	17	100 (81.4–100)	100 (97.1–100)	100 (0–100)

Median of percentages, minimum and maximum values in parentheses.

When the students were asked whether selected items were derived (or not) from the forest, they unanimously answered “Yes” when “firewood” and “wooden house” photos were selected. In the case of “medicinal plant” and “lamba” (a traditional cloth dress), percentages were more variable (Tables 4 and 5). No differences in responses were found by training status or school location.

**Table 4:** Descriptive statistics for responses to medicinal plant, classrooms are categorized by training status or location.

*Medicinal plant comes from the forest*?				
*Training?*	*#Schools*	*Median*	*Mean*	*Min-Max*
Yes	10	85.30	70.67	55.6–100
No	10	100.00	92.03	2.4–100
*City school*?	*#Schools*	*Median*	*Mean*	*Min-Max*
Yes	9	94.29	70.04	2.4–100
No	11	100	90.60	55.6–100

No significant differences were found.

**Table 5:** Descriptive statistics for responses to “cloth dress”, classrooms are categorized by training status or location.

*“Cloth dress” comes from the forest*				
*Training?*	*#Schools*	*Median*	*Mean*	*Min-Max*
Yes	15	24.00	32.24	0–100
No	11	62.5	56.08	5.3–94.6
*City school*?	*#Schools*	*Median*	*Mean*	*Min-Max*
Yes	9	24	38.26	5.3–100
No	17	30.56	44.48	0–85.7

No significant differences were found.

### Water section

When students were asked whether different water sources were “safe to drink”, boiled water and water from a forest stream scored high, whereas water from rice paddies scored low. No significant differences were found when respondents from city schools were compared to respondents from village schools. When training status was considered, classrooms with “training” displayed higher percentages affirming forest stream water was okay to drink, compared to classrooms with “no training” (Wilcoxon Mann-Whitney test, z = -2.3254, *p* = 0.02).

When students were asked what sources of water *they* drink at home, boiled water showed the highest percentages. Although no significant differences were found when classrooms were classified by training status, there were differences in the source of water used between city and village schools: students from the city rely more on wells and purified water than those from villages, who use, sometimes, the local river as source of drinkable water (river water, Wilcoxon Mann-Whitney test, z = -2.02, *p* = 0.04; water from well, Wilcoxon Mann-Whitney test, z = 2.44, *p* = 0.01; purified water, Wilcoxon Mann-Whitney test, z = 3.23, *p* = 0.001) (Tables 6 and 7).

**Table 6:** Sources of water acceptable to drink and sources used at home.

*Drinkable?*	*Median*	*Mean*	*At home*?	*Median*	*Mean*
boiled water	100	98.03	boiled water	97.77	88.46
water from forest stream	100	93.2	water from a well	11.9	37.21
water from a well	81.08	64.27	water from local river	4.55	23.95
water from rice paddies	0	7.07	purified water	2.86	16.15

**Table 7:** Sources of water acceptable to drink and sources used at home, with responses classified by school location.

*Villages*			*City*		
*At home*?	*Median*	*Mean*	*At home*?	*Median*	*Mean*
boiled	100	88.44	boiled	95.22	88.49
well	2.99	25.59	well	53.38	55.6
local river	5.97	34.5	local river	0	7.25
purified	2.33	5.87	purified	34.83	32.39

Finally, when students were asked about whether humans and fish needed clean water for survival, responses were dissimilar: although there is a general agreement that humans do need clean water, students from classrooms with “training” responded more affirmatively than students from classrooms with “no training” when it came to fish needing clean water (Wilcoxon Mann-Whitney test, z = 2.31, *p* = 0.02) (Table 8). When students were asked whether it was okay to dispose of garbage in the river, and whether it was acceptable to throw poison in the water to catch fish, percentages were consistently low across classrooms. No significant differences were found when classrooms were compared by training status or location (Table 8).

**Table 8:** Responses to the questions of whether human and fish need clean water to survive, and whether garbage and poison are okay.

*Training*	*#Schools*	*Clean water for fish*	*Clean water for humans*	*Garbage okay*	*Poison okay*
yes	18	97.73 (0–100)	100 (97.1–100)	0 (0–100)	0 (0–100)
no	13	54.55 (0–97.7)	100 (95.5–100)	0 (0–100)	4.76 (0–100)
*City*	*#Schools*	*Clean water for fish*	*Clean water for humans*	*Garbage okay*	*Poison okay*
yes	12	59.76 (4–100)	100 (0–100)	0 (0–100)	2.27 (0–100)
no	19	95 (0–100)	100 (95.5–100)	0 (0–100)	0 (0–100)

Classrooms classified by training status and location.

## Discussion

The students in our study had knowledge about the environment, but it was fragmented, inconsistent and lacked some basic distinctions. For instance, a relatively large portion of students agreed with the statement that wild animals could be kept in households, and many students agreed with the sentence that a lemur did not need the forest for survival. The familiarity with local biodiversity was partly lacking. For instance, indri lemurs are present in Anjanaharibe Sud Special Reserve, in the Andapa District and not far away from the sampled schools (between 15 and 100Km). The fact that students generally failed to identify the indri lemur suggests that they are not aware of the biodiversity present in the local protected forests of the SAVA region. Ring-tailed lemurs were more easily identifiable, despite the greater geographic distance from SAVA, suggesting that the students learned of this iconic lemur from its frequent depiction in books, movies, social media, and other kinds of advertisement.

Regarding their perceptions about the forest, students from village schools tended to value forests and rice paddies equivalently. This is interesting because communities living in small villages are more reliant on rice cultivation as a subsistence agricultural practice. Whether students have a rationale for valuing forest like rice paddies, or whether students guessed that was the answer that the surveyor was “hoping for” is unclear. Furthermore, although the association between forests and wood-based products such as firewood was clear, there was more disparity in responses associated with other products, such as medicinal plants.

Finally, most students identified boiled water and forest streams as sources of drinkable water. Though it can be argued that boiled water is a safe option, the quality of forest streams may be variable depending on the location and exposure to potential pollutants. Clean water for humans was acknowledged, but the connection between clean water for fish, and the importance of the quality of water in ecosystems (e.g., acceptable to use poison to catch fish) was less apparent, at least for a portion of the surveyed students.

### Survey setting

For this survey we tried to minimize ambiguity from the questions, allow students to answer several inquiries in a timely manner, and make it easy for the students to respond, without reliance on writing skills. At the same time, we understand that the students were given a simple choice of “yes” to respond to complex questions, e.g., forest is as valuable as a rice paddy. Thus, classroom’s percentages may not reflect the actual students’ perceptions. We take the responses from these surveys as preliminary, and potentially informative for future inquiries through focus-group surveys.

### Are teacher trainings enough?

Trained teachers are encouraged, but not obligated, to incorporate EE content in the curricula. Therefore, tracking and interviewing individual teachers would be the only way to determine how much, if any, of the EE material was incorporated to their curricula. Such surveys, however, would be logistically difficult to carry out, especially given the high teacher turnover in the SAVA region (see below). Generally, teacher trainings have been successful at Ivoloina, a zoological facility and forest station in eastern Madagascar, where students have access to wildlife as they learn about the environment [9]. Teacher trainings are expected to be more effective in areas with natural environments, where students can undergo experiential learning, but also in areas where teachers have a stable and long-term presence in local schools. The SAVA region is auspicious on the former, and less promising on the latter. SAVA encompasses one of the largest concentrations of remaining forests in Madagascar, including Marojejy National Park, Anjanaharibe Sud Special Reserve, COMATSA (a forest corridor connecting Marojejy, Anjanaharibe Sud and Tsaratanana Nature Reserve to the north), the northern portion of Makira Natural Park, Makirovana-Tsihomanaomby complex, and Masoala National Park. The DLC-SAVA has supported school visits to local reserves and National Parks, and is in the process of integrating those visits with dynamic engaging activities at schools, before, during and after expeditions. However, low education levels and high teacher turnover also characterize areas of the SAVA region. The teachers’ frequent migration may be partly explained by generally unsatisfactory and uncertain salary compensations, added to the fact that the costs of living in SAVA, being a vanilla-producing region, are much greater than in other regions across Madagascar. In fact, many teachers are not contracted full-time by the Government and rely on parent associations for salary. Likely as a result of difficulties in recruiting teachers, the teachers’ qualifications, in turn, seem to have been relaxed over time. A lack of specialized training for teachers may constrain their ability to develop and implement curricula content required in some of the EE programs. An additional monitoring of teachers’ educational background, long-term presence, and plans to remain in the area may be necessary as a precondition for recruitment in future trainings, to maximize the chance that EE content may be implemented in SAVA schools.

Although the role of NGOs is critical for implementing EE programs in Madagascar and worldwide, it is also undeniable that in terms of financial support and scale, they cannot substitute efforts by the government [9]. Education reform is needed to bridge generational gaps in education and pedagogical techniques (that is also evident in western countries) [17,18]. As put forth by Hardman et al. [6] p. 670 “…Comparative research shows that teacher reform needs to combine the culturally or nationally unique with what is universal in classroom pedagogy if internationally driven reforms to teacher education are to be embedded in the classroom reforms…”

### Recommendations

Both the feeble distinction between domesticated and wild animals and the lack of familiarity with the local biodiversity call for greater action. The discussion about wildlife could be incorporated in the context of a widespread problem, the illegal pet trade in Madagascar. “Pet” lemurs are sometimes used as educational tools by school Directors, a money source by private parties who profit from pictures of tourists holding lemurs [MBB pers. obs.], but also as animal “companions” for children. The problems that derive from keeping wildlife in captivity are well reported and include the potential for disease transmission, injuries due to aggression, and, the inhumane and inadequate conditions wildlife must endure [19]. Environmental education activities should address the difference between domesticated and wild animals, why lemurs cannot survive without a forest and why wildlife is important to forest health, while exemplifying the diet and habits of wildlife and integrating wildlife education with other components of the environment in a more dynamic and active way.

A second set of activities could refer to consumption of wildlife. Taboos (*fady* in Malagasy) against wildlife consumption have a cultural basis and vary across regions in Madagascar. Superimposed with cultural practices, there are Government laws that regulate the use and management of natural resources, including selected wildlife. Unfortunately, the lack of information about cultural vs. legal taboos can mislead people who commit illegal activities inadvertently: For example, in some areas in eastern Madagascar, 60 to 90% of respondents in remote villages believed they could legally eat lemurs, despite the fact that lemurs have had legally protected status for over 50 years [7,20]. As Keane et al. [20] point out, laws can be effective insofar as they are known and understood by the population under their jurisdiction. Although posters against lemur consumption, containing explicit imagery may help to raise awareness, they do not provide a rationale powerful enough to be integrated as practice. That rationale should be constructed rather than imposed through prohibition and punishment. We recommend developing EE activities that take into consideration *narratives* and stories about local and regional taboos, with students enquiring other members of the communities and learning more about the natural history of the wildlife around them.

The comparable value of forest and rice paddies, reflected in some of the survey responses, highlights an important point about perceptions concerning land use and management [21,22]. EE activities can be designed to address the environmental impact of certain practices, such as rice cultivation, vanilla plantations, or controlled forest extraction on soil composition, erosion, water conservation, and other ecological matters. Land productivity, but also biodiversity content can be modeled under a variety of scenarios, at different spatial and temporal scales. The introduction of vanilla plantations into the discussion is pertinent in the SAVA region, where money fever is driving the local economy, prompting community members to invest heavily in vanilla despite great associated risks. Interdisciplinary research teams are currently investigating the consequences of vanilla production in the economy, among social relations, and across biodiversity levels [23]. This information could be fed back to school students by proposing realistic scenarios and discussing problem-solving solutions.

**Fig 1:**
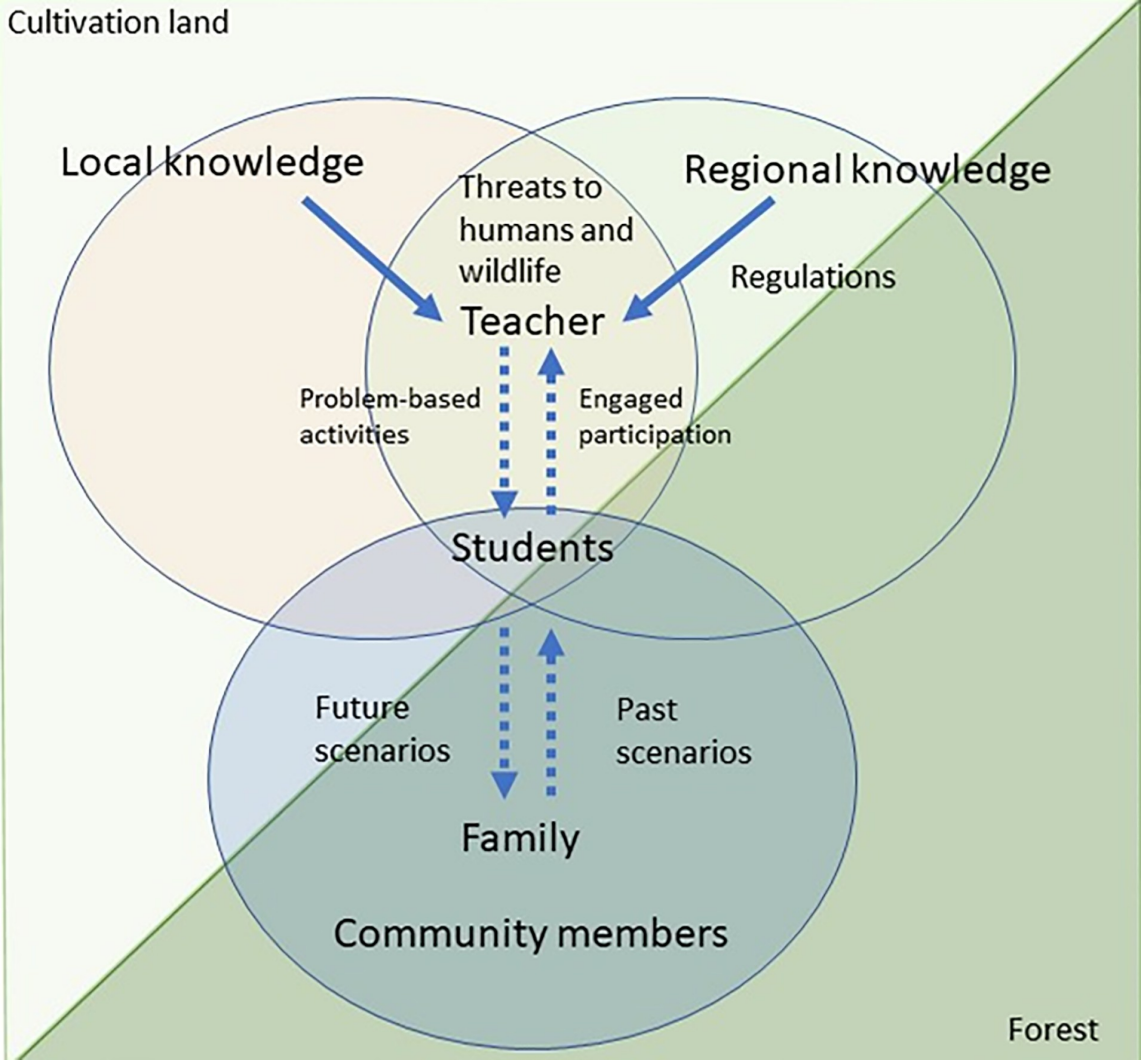
Proposed conglomerate of information, directional flow, participants and scenarios involved in environmental education activities.

Furthermore, we would like to point out that awareness and knowledge regarding local biodiversity, conservation risks and regulations should be the baseline from which to discuss local environmental problems, and shape attitudes towards protection (Fig 1). We acknowledge that teachers may need additional assistance from researchers and trained educators not only to develop, but to implement new activities. For further monitoring, we recommend the use of oral surveys, to avoid discrepancies due to literacy levels across students. Additionally, group activities (to avoid biased responses towards more outgoing students), or interest-based surveys targeting students interested in the environment, can complement more general classroom surveys. A more personalized approach, by engaging a subset of students who want to participate in environmental activities, may be relevant in areas where resources and qualified staff are limited.

Finally, although we support the use of Manuals and other materials used for EE programs targeting teachers, we recognize the importance of discussing local problems that students can relate to in daily life, by focusing on local biodiversity and habitat threats (as opposed to more global and less relatable threats), by incorporating local knowledge, and by encouraging students to ask family members and others in the community to participate in classroom dialogue. One last note to NGOs interested in EE programs, like DLC-SAVA, is to propose monitoring activities as part of the long-term vision, and account for their expenses in budgetary discussions. Mandatory conversations with donors about the need for evaluation strategies to assess the progress of a given program, for instance, may help overcome financial burdens. More importantly, they will urge researchers and educators in charge of those programs to design and implement appropriate supervision plans.

## Supporting information

S1 Appendix Classroom-based Survey questionnaire in English. (DOCX)
