# Supplementary material for: Back to basics: Gaps in baseline data call for revisiting an environmental education program in the SAVA region, Madagascar
Source: PLoS One. 2020 Apr 21;15(4):e0231822. doi: 10.1371/journal.pone.0231822 (PMC7173847; doi:10.1371/journal.pone.0231822)
Supplement: S1 Appendix — (DOCX) [file pone.0231822.s001.docx]

**Classroom-based Survey-English version**

**Survey Objective**

The objective of this survey is to gather baseline quantitative data on students’ perceptions and knowledge of their environment. This survey seeks to compare the environmental knowledge and perspectives of students whose teachers have received environmental education training to those whose teachers have not received training. The data recorded from this survey will inform environmental education programs and outreach, and will serve as baseline data for monitoring and evaluating these education programs.

**Survey Instruction**

Before administering this survey, the surveyor will be trained in its administration. The surveyor must take precautions so as not to divulge any verbal (including intonations), non-verbal, emotive hints about any of the answers.

Surveyor will dictate the following questions, in a manner that is slow, easy to understand, and unbiased. At least one other surveyor should be present in the room to count / estimate hands raised for each question and record class-wide answers. Recruit the help of a teacher to count hands as well.

Surveyor should pause after every question asked and tally the hands. Please instruct the children to keep their hands raised until a count has been tallied.

At each school, prior to survey administration, record the following:

-School location, school name

-Whether teacher participated in a Teacher Training program

-Time and date of survey administration

-Number of students present in the class

-Name of project staff that helped administer the survey

**Survey Materials**

*Pictures :

-Animal Pictures: chicken, dog, tenrec, mouse lemur, ring-tailed lemur, indri lemur, and brown lemur

-Forest Pictures: a burnt (disturbed) forest, a pristine (undisturbed) forest

-Water: forest stream, river next to a village, well, boiled water, water from rice paddy, purified water

*Bags for photo selections

*Table and answer sheets for the survey administrator and observers to record their observations

________BEGIN SURVEY_________________________________________________

**Surveyor to the class:**

“We are going to ask you some questions about nature, to see what you know. You are not required to participate in this survey and may leave the room at any point you wish. You will not be in trouble if you choose to leave.

Please answer the following questions as honestly as possible, without looking at your classmates if you don't know the answers.

We are going to ask you a question, and when you think you know the answer, raise your hand. Only raise your hand if you are sure that your answer is right, and leave your hand raised until instructed by the surveyor to put them down.”

**Animals:**

For this section, the surveyor will select three children to come up and each pick a photo from a bag, where all the animal photos will be located. Each photo will have an accompanying set of questions on the back of the photo. It is **imperative** that the surveyor and observers note which animals were selected from the bag.

[Show picture of Chicken]


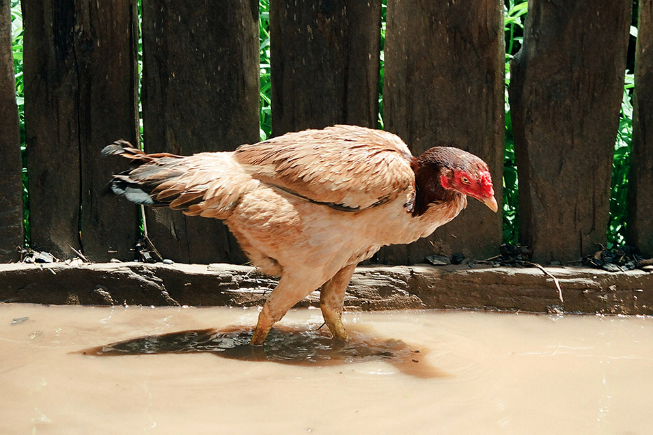


1. Is this animal a duck? Raise your hand if you think YES. [pause]

2. Is this animal a chicken? Raise your hand if you think YES. [pause]

3. Is this animal most often found in the forest? Raise your hand if you think YES.

4. Is this animal most often found in the village? Raise your hand if you think YES.

5. Is it acceptable to keep this animal in the house? Raise your hand if you think YES.

6. Have you seen this animal in the forest or in the village? Raise your hands if YES.

7. Is it taboo [*“fady”*] to eat this animal? Raise your hand if you think YES.

[Show picture of tenrec]


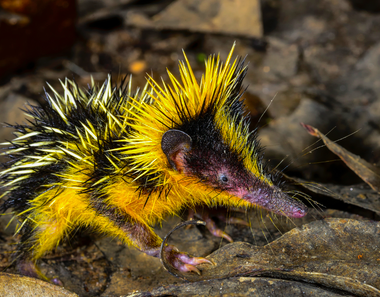


1. Is this animal a tenrec? Raise your hand if you think YES.

2. Is this animal a rat? Raise your hand if you think YES.

3. Is this animal most often found in a rice paddy? Raise your hand if you think YES.

4. Is this animal most often found in the forest? Raise your hand if you think YES.

5. Is it acceptable to keep this animal in the house? Raise your hand if you think YES.

6. Have you seen this animal in the forest or in the village? Raise your hand if YES.

7. Is it taboo to eat this animal? Raise your hand if you think YES.

[Show picture of dog]


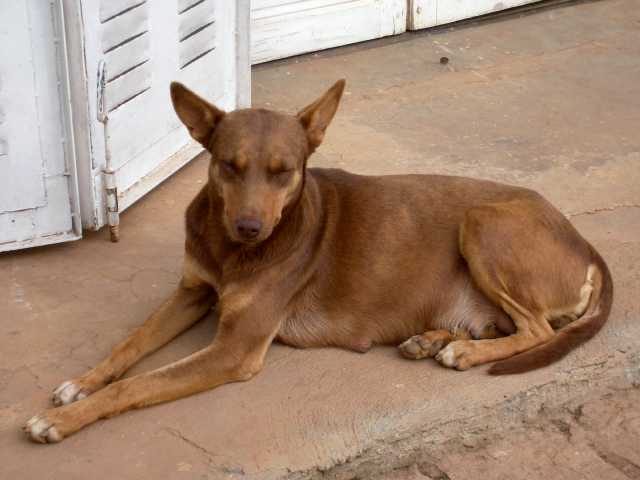


1. Is this animal a fossa? Raise your hand if you think YES.

2. Is this animal a dog? Raise your hand if you think YES.

3. Is this animal most often found in the forest? Raise your hand if you think YES.

4. Is this animal most often found in the village? Raise your hand if you think YES.

5. Is it acceptable to keep this animal in the house? Raise your hand if you think YES.

6. Have you seen this animal in the forest or in the village? Raise your hand if YES.

7. Is it taboo to eat this animal? Raise your hand if you think YES.

[Show picture of mouse lemur, local name tsitsihy]


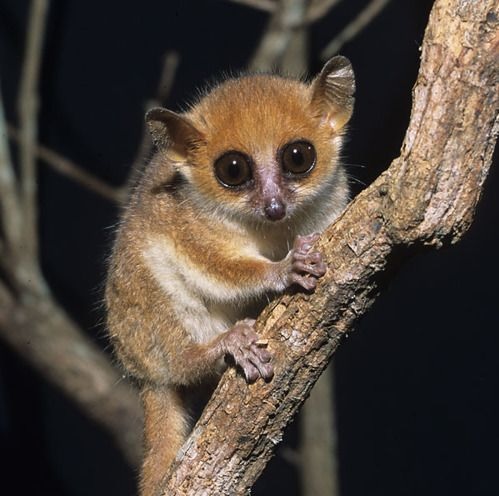


1. Is this animal a mouse lemur [tsitsihy]? Raise your hand if you think YES.

2. Is this animal an indri [babakoto]? Raise your hand if you think YES.

3. Is this animal most often found in the village? Raise your hand if you think YES.

4. Is this animal most often found in the forest? Raise your hand if you think YES.

5. Is it acceptable to keep this animal in the house? Raise your hand if you think YES. 6. Have you seen this animal in the forest or in the village? Raise your hand if YES.

7. Is it taboo to eat this animal? Raise your hand if you think YES.

[Show picture of ring-tailed lemur, local name maki]


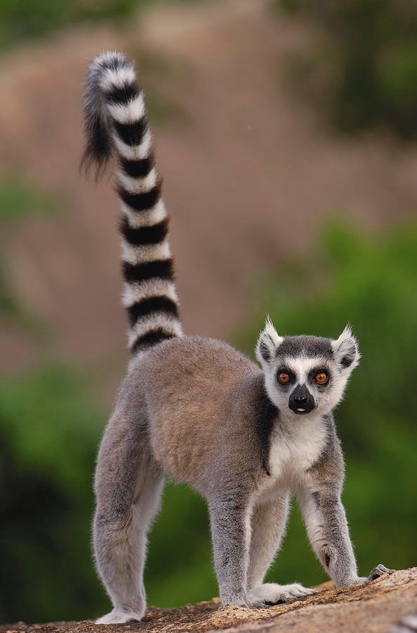


1. Is this animal a ring-tailed lemur [maki]? Raise your hand if you think YES.

2. Is this animal a cat? Raise your hand if you think YES.

3. Is this animal most often found in the village? Raise your hand if you think YES.

4. Is this animal most often found in the forest? Raise your hand if you think YES.

5. Is it acceptable to keep this animal in the house? Raise your hand if you think YES.

6. Have you seen this animal in the forest or in the village? Raise your hand if YES.

7. Is it taboo to eat this animal? Raise your hand if you think YES.

[Show picture of indri lemur, local name babakoto]


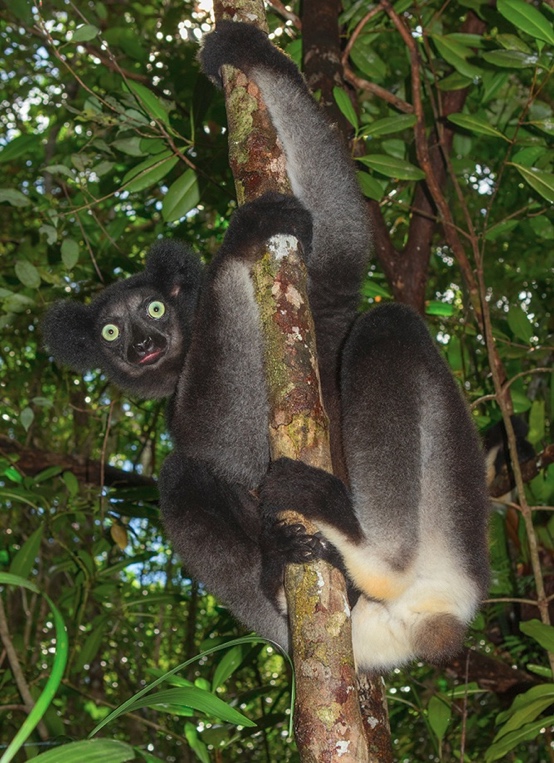


1. Is this animal a mouse lemur [tsitsihy]? Raise your hand if you think YES.

2. Is this animal an indri [babakoto]? Raise your hand if you think YES.

3. Is this animal most often found in the village? Raise your hand if you think YES.

4. Is this animal most often found in the forest? Raise your hand if you think YES.

5. Is it acceptable to keep this animal in the house? Raise your hand if you think YES.

6. Have you seen this animal in the forest or in the village? Raise your hand if YES.

7. Is it taboo to eat this animal? Raise your hand if you think YES.

[Show picture of brown lemur, local name ankomba]


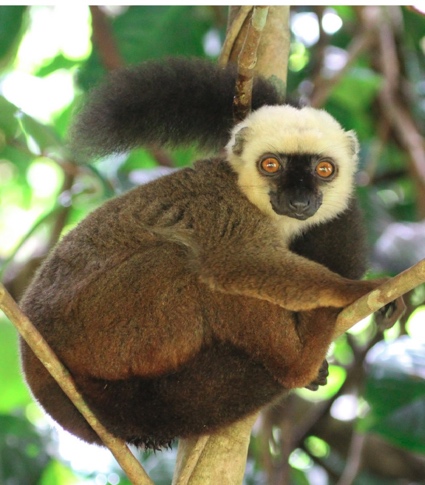


1. Is this animal a brown lemur [ankomba]? Raise your hand if you think YES.

2. Is this animal a fossa? Raise your hand if you think YES.

3. Is this animal most often found in the village? Raise your hand if you think YES.

4. Is this animal most often found in the forest? Raise your hand if you think YES.

5. Is it okay to keep this animal in the house? Raise your hand if you think YES.

6. Have you seen this animal in the forest or in the village? Raise your hand if YES.

7. Is it taboo to eat this animal? Raise your hand if you think YES.

**Trees:**


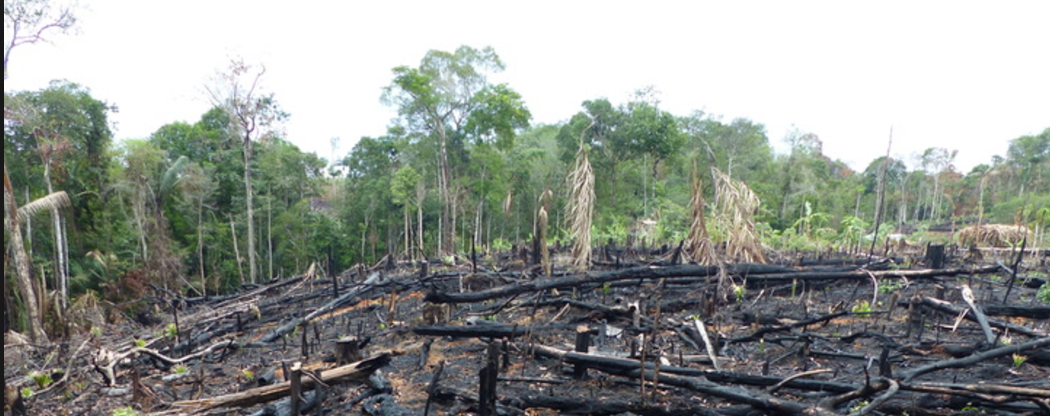


1. [Show a picture of a burnt, cut down forest] Is this forest good [tsara]? If you think YES, raise your hand.
2. [Show a picture of a pristine forest] Is this forest good [tsara]? If you think YES, raise your hand.
3. [Have children select 3 photos from the tree section bag]. When I show you a picture, if you think that it comes from the forest, please raise your hand.

[Show Medicinal Plant picture] Can you find this in the forest? If YES, raise hand.

[Show House picture] Can you get this from the forest? If YES, raise your hand.

[Show Cell Phone picture] Can you get this from the forest? If YES, raise your hand.

[Show Firewood picture] Can you get this from the forest? If YES, raise your hand.

[Show Cloth dress [lamba] picture ] Can you get this from the forest? If YES, raise your hand.

1. Does the forest take a long time to grow? If YES, raise your hand.
2. [Show picture of a lemur] Can this live without the forest? If YES, raise your hand.
3. Does a tree come from a mattress? If YES, raise your hand.
4. Does a tree come from a seed? If YES, raise your hand.
5. Is a forest as important as a rice paddy? If YES, raise your hand.

**Water:**

1. [Show picture of forest stream] This is a stream in a forest. Is it safe to drink this? Raise your hands if you think YES.

2. [Show picture of well]. This is a well. Is it good to drink this water? Raise your hands if you think YES.

3. [Show picture of boiled water] This water is boiled. Is it safe to drink this water? Raise your hands if you think YES.

4. [Show picture of rice paddy water] This is water from a rice paddy. If you drink this water, please raise your hand.

5. Do fish need clean water to live? Raise your hands if you think YES.

6. Do people need clean water to live? Raise your hand if you think YES.

7. Is it safe to throw garbage [fako] in the river? If YES, raise your hand.

8. Is it safe to throw poison in the river to catch fish? If YES, raise your hand.

9. I am going to show you pictures of different sources of water. If you drink this at home, please raise your hand. You may raise your hand for more than one.

[River water picture]

[Boiled water picture]

[Well water picture]

[Purified water picture]

________END SURVEY_________________________________

Thank the class for their honesty, patience, and participation.
